# Physics-informed W-Net GAN for the direct stochastic inversion of fullstack seismic data into facies models

**DOI:** 10.1038/s41598-024-55683-5

**Published:** 2024-03-01

**Authors:** Roberto Miele, Leonardo Azevedo

**Affiliations:** CERENA, Department of Mineral and Energy Resources Engineering, Instituto Superior Técnico, Av. Rovisco Pais 1, 1049-001 Lisbon, Portugal

**Keywords:** Geophysics, Carbon capture and storage, Energy storage, Fossil fuels, Renewable energy, Hydrology, Solid Earth sciences

## Abstract

Predicting the subsurface spatial distribution of geological facies from fullstack geophysical data is a main step in the geo-modeling workflow for energy exploration and environmental tasks and requires solving an inverse problem. Generative adversarial networks (GANs) have shown great potential for geologically accurate probabilistic inverse modeling, but existing methods require multiple sequential steps and do not account for the spatial uncertainty of facies-dependent continuous properties, linking the facies to the observed geophysical data. This can lead to biased predictions of facies distributions and inaccurate quantification of the associated uncertainty. To overcome these limitations, we propose a GAN able to learn the physics-based mapping between facies and seismic domains, while accounting for the spatial uncertainty of such facies-dependent properties. During its adversarial training, the network reads the observed geophysical data, providing solutions to the inverse problems directly in a single step. The method is demonstrated on 2-D examples, using both synthetic and real data from the Norne field (Norwegian North Sea). The results show that the trained GAN can model facies patterns matching the spatial continuity patterns observed in the training images, fitting the observed geophysical data, and with a variability proportional to the spatial uncertainty of the facies-dependent properties.

## Introduction

Geological facies models are two- or three-dimensional numerical representations of the spatial distribution of litho-fluid classes (i.e., geological formations sharing the same characteristics in terms of mineral and saturating fluids content) in the subsurface^1^. The accurate prediction of the spatial distribution of facies at targeted depths is required in many studies related to geo-energy and environmental applications. These types of models are key pieces of information, for example, to characterize groundwater systems, geothermal or hydrocarbon reservoirs, or to assess the capacity of carbon dioxide geo-storage^2–5^.

As direct observations of deep geological formations (i.e., well-logs) are generally scarce and provide spatially limited information, geophysical measurements can be used as indirect measurements of the subsurface geological properties for the prediction of the spatial distribution of facies and rock properties. This goal is normally achieved by finding the solutions to an ill-posed and highly nonlinear geophysical inverse problem accounting for nonunique solutions, due to noise in the recorded geophysical data, errors in the models assumed to describe the subsurface properties’ distributions, and approximations to the geophysical model (i.e., elastic wave propagation) under investigation^1,6–8^. When fullstack seismic reflection data is inverted, the subsurface acoustic impedance (${\mathbf{I}}_{\mathbf{p}}$) can be used as the facies-dependent elastic property to model the relationship between facies and observed seismic data (${\mathbf{d}}_{\mathbf{o}\mathbf{b}\mathbf{s}}$) domains^7,9^. The prediction of facies from fullstack seismic data can be summarized as follows: synthetic seismic data is calculated from a facies pattern using the collocated ${\mathbf{I}}_{\mathbf{P}}$ distribution; the solutions to the inverse problem are the facies patterns that minimize the misfit between synthetic seismic data and ${\mathbf{d}}_{\mathbf{o}\mathbf{b}\mathbf{s}}$^6,10–12^. Although a number of deterministic seismic inversion methodologies exist^6,10^, these approaches always predict smooth representations of the subsurface (i.e., the predictions are not able to account for small-scale geological variability) and have limited capabilities to represent the uncertainty of the retrieved solutions. On the other hand, stochastic seismic inversion algorithms can predict a distribution of possible solutions to the inverse problem, allowing uncertainty assessment ^7,12,13^. These algorithms generally rely on Bayesian inversion^14–16^, stochastic optimization^11,12,17–19^, or Markov chain Monte Carlo (McMC) sampling^20–22^ of the facies model parameters that better fit the observed data.

In seismic inversion algorithms, representing uncertainties of facies distributions is key to unbiased and accurate predictions^3^. Common facies modeling techniques used in seismic inversion are geostatistical simulation methods based on the reproduction of two-points (variogram)^7^ or multiple-point statistics (MPS)^23,24^, which parameters can be optimized to find the desired inversion problem solutions. Variogram-based simulation methods are widely adopted for the reproduction of petrophysical or elastic properties (e.g., ${\mathbf{I}}_{\mathbf{P}}$) continuity patterns, which are generally modelled from direct observations (e.g., well-log data). Nonetheless, they fail in reproducing high-order spatial statistics of depositional patterns, such as channels and lobes^7^. Contrarily, MPS-based algorithms generate realizations by directly sampling the spatial patterns stored in a training image, which represents the prior knowledge on the subsurface geology (e.g., a geological conceptual model)^3,25^. Nonetheless, geophysical inversion using such methods require to optimize a large number of parameters (i.e., finding a distribution of subsurface values that solves the inverse problem, for each location of the inversion grid). This can lead to demanding or prohibitive computational costs^26^. Recently, various deep learning (DL)^27^ methods have been proposed as generators of spatial geological patterns, including facies in geophysical inversion^12,28–35^. In particular, generative adversarial networks (GANs)^36^ have been proven to be particularly suitable in complex and geologically accurate facies modeling. GANs are composed of two networks, a generator and a discriminator. The generator is trained to map high-dimensional images (e.g., the facies patterns) from a low-dimensional distribution, reproducing spatial features in the training data set. The discriminator is trained to distinguish between real (i.e., observed) and generated data. This dual, adversarial, training process refines the generator's output to fool the discriminator; a trained generator is hence able to model facies patterns into fewer latent variables, maintaining modeling performances that are comparable to those of MPS-based simulations^26,29,31,37–39^. The low-dimensional encoded latent space can be explored by means of an inference method (e.g., McMC^26,40^ or variational inference^33,41,42^) to retrieve the facies spatial patterns that minimize the geophysical data misfit (i.e., the residuals between observed and predicted synthetic seismic data). Despite the successful demonstration of this technique in synthetic cases ^26,33,40–42^, the model parameters are optimized without accounting for the uncertainty affecting the facies-dependent elastic properties (e.g., ${\mathbf{I}}_{\mathbf{P}}$). The solutions retrieved are hence dependent on the facies’ uncertainty alone, which can lead to inaccurate predictions (i.e., unrealistic from a geological perspective). Moreover, despite the GANs ability to reduce the dimensionality of a geophysical inversion, each new application is likely to require another training of the network (i.e., to represent a new geological prior), followed by the actual geophysical inversion in a second step. This approach can still represent a time-consuming and computationally expensive process for complex geological scenarios.

The work proposed herein introduces a GAN architecture to directly model realistic facies patterns conditioned on ${\mathbf{d}}_{\mathbf{o}\mathbf{b}\mathbf{s}}$, given the facies-dependent ${\mathbf{I}}_{\mathbf{P}}$ uncertainty, in a single training phase. The network leverages on a specific discriminator architecture to learn the prior facies patterns and their physics-based correlation to the seismic data domain. For this purpose, we use a multivariate distribution defined by a set of subsurface facies images, paired with a nested distribution of corresponding fullstack seismic data responses. The latter are calculated from ${\mathbf{I}}_{\mathbf{P}}$ geostatistical realizations locally conditioned on the facies sample, honoring the facies-dependent experimental data distributions and spatial uncertainty^43^. The discriminator encodes both facies and seismic data features into three individual scores, representing marginal and joint probabilities between the two domains. The discriminator evaluates the facies generated by an unconditional generative network coupled with ${\mathbf{d}}_{\mathbf{o}\mathbf{b}\mathbf{s}}$, in terms of facies geological accuracy and fitting to the seismic data. The inversion is carried out by training the generator to simultaneously maximize these two scores. Such adversarial training allows to finally obtain a generator able to reproduce several facies patterns fitting ${\mathbf{d}}_{\mathbf{o}\mathbf{b}\mathbf{s}}$, while approximating a posterior distribution. Given the specific three-branched, W-shaped architecture of the discriminator, which reads two separate inputs and generates three output scores, we refer to this method as W-Net GAN.

The proposed methodology is first evaluated on 2-D synthetic application example, using a test data set integrating scenarios not reproduced by the prior training data. The method is further adopted for the inversion of a real 2-D seismic section acquired in the Norwegian Sea, in the Norne oil field. In all the application examples our results show that the trained generator can reproduce a probabilistic map corresponding to the facies targets within the assumed ${\mathbf{I}}_{\mathbf{P}}$ uncertainty range, while generating facies distributions indistinguishable from those of the training data set. The performances of the network suggest that the proposed approach can be used within the geo-modeling workflow to create subsurface numerical models integrating the a priori geological knowledge, data statistics and geophysical forward model.

## Method

### Training data set

The proposed seismic inversion technique is based on training the W-Net GAN with a training data set representing the prior knowledge on the facies distributions (i.e., the expected geological setting) and the corresponding fullstack seismic reflection data. The latter is obtained by modeling the facies-dependent ${\mathbf{I}}_{\mathbf{P}}$ distributions and its spatial uncertainty (e.g., from existing direct observations such as well-log data). The representation of the prior knowledge in the training data set is defined within the inversion grid lateral extension (i.e., for the fullstack seismic volume), that of the ${\mathbf{d}}_{\mathbf{o}\mathbf{b}\mathbf{s}}$ considered in the case study^7^.

To represent the subsurface geology, we use a set of $N$ 2-D facies generated with geostatistical simulations conditioned to MPS, sampling the patterns from a conceptual geological model. Alternative simulation methods could be used (i.e., physics-based models). Based on the existing well-log data, we then model a variogram representing the spatial continuity patterns of ${\mathbf{I}}_{\mathbf{P}}$ in each facies. Conditioned to each MPS facies realization, we generate a set of $M$ geostatistical simulations of ${\mathbf{I}}_{\mathbf{P}}$ using the direct sequential simulation (DSS) method^43,44^, honoring locally the expected facies-dependent ${\mathbf{I}}_{\mathbf{P}}$ values distribution and modelled continuity patterns. In cases where no direct observations are available a priori information from analogue geological settings might be used. Therefore, the resulting ensemble of realizations represents a nested set of equiprobable spatial distributions of ${\mathbf{I}}_{\mathbf{P}}$, conditioned on the potentially available direct measurements, spatial continuity pattern and facies distribution. We finally model the correlation between ${\mathbf{I}}_{\mathbf{P}}$ and fullstack seismic reflection data. First, we calculate the normal-incidence reflectivity coefficients ($\mathbf{r}$) from:

1 $$\mathbf{r}=\frac{{{\mathbf{I}}_{\mathbf{P}}}_{i+1}-{{\mathbf{I}}_{\mathbf{P}}}_{i}}{{{\mathbf{I}}_{\mathbf{P}}}_{i+1}+{{\mathbf{I}}_{\mathbf{P}}}_{i}},$$

where $i$ represents the sample above the interface where the reflection coefficient is being calculated and $i+1$ represents the sample below. Then, we convolve the reflection coefficients with a known source wavelet, assumed to be the one that best describes the observed seismic data^10^. The discussion about wavelet estimation methods is out of the scope of this work. This operation is repeated for each ${\mathbf{I}}_{\mathbf{P}}$ realization, for each facies pattern in the training dataset. Therefore, we obtain a nested set of *M* seismic reflection data corresponding to the *N* facies pattern, representing the ${\mathbf{I}}_{\mathbf{P}}$ spatial uncertainty indirectly, through different seismic realizations. During the training process, a single sample of the training data set will be defined by a facies pattern image and its corresponding seismic data, randomly sampled from the nested set.

### W-Net GAN architecture

**Figure 1:**
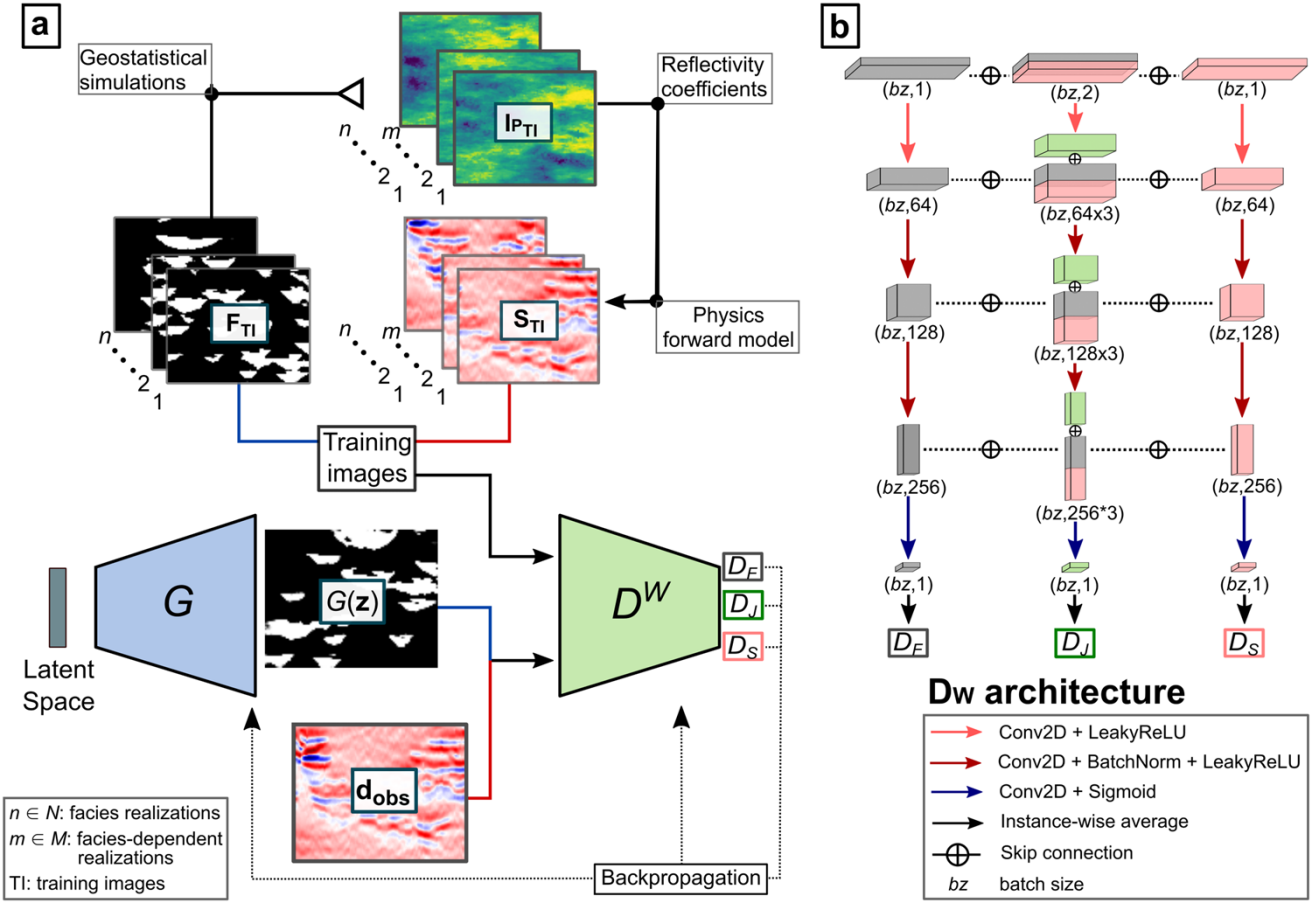
Schematic representation of the proposed seismic inversion through the W-Net GAN; (**a**) inversion algorithm, (**b**) details about the architecture of the W-Net GAN discriminator (${{\text{D}}}^{{\text{W}}}$).

The W-Net GAN (Fig. 1a) uses a generative network ($G$) to map a random latent vector ($\mathbf{z}$) into a 2-D facies model. Its architecture is composed of a sequence of four transposed convolutional hidden layers, using batch normalization (BatchNorm) and leaky rectified linear units (LeakyReLU) activation functions to decode $\mathbf{z}$. The output layer is another transposed convolutional layer with hyperbolic tangent (Tanh) as activation function, to finally define binary facies images, classified as − 1 and 1 (respectively shale and sand classes).

The discriminator (${D}^{W}$ in Fig. 1a and b) is composed of three distinguished convolutional neural networks, or branches, each with four layers. This architecture is designed to read simultaneously the facies and seismic data as input using two branches (‘external’) and encode the information from the two domains into three likelihood scores: one for the facies features (${D}_{F}$), one for the seismic data features (${D}_{S}$) and another for their joint distribution (${D}_{J}$). The first two scores are independently generated by the two external branches. The output resulting from each hidden layer of the external branches is also concatenated and transferred to a central branch by means of skip connections (Fig. 1b). The central branch evaluates the ${D}_{J}$ score by encoding the joint features. The set of skip connections reinforce the spatial characteristics of facies and seismic domains at different spatial scales. Analogous to $G$, the hidden layers’ use BatchNorm and LeakyReLU activation functions. The output layer of each branch accounts for a convolutional operation and a sigmoid activation function. The outputs are score matrices which are then averaged (i.e., instancewise average in Fig. 1b). The proposed architecture can be adapted to different image sizes and complexities. We used different kernel sizes, padding and stride parameters for both $G$ and ${D}^{W}$, to adapt the network to the synthetic and real case application examples shown below. A detailed representation of the architectures used in this work is presented in the Supplementary Information. Although the W-Net GAN proposed here predicts a binary facies distributions, the architecture can be further adjusted to produce a larger number of facies (e.g., using one hot encoding^30^).

### Seismic inversion with W-Net GAN

The proposed seismic inversion workflow is illustrated in Fig. 1a. After representing the prior information into a training data set, the seismic data inversion is carried out directly through the training of the W-Net GAN, conditioning the network optimization on both training data and ${\mathbf{d}}_{\mathbf{o}\mathbf{b}\mathbf{s}}$. As the correlation between facies and seismic data is modelled by the physics forward model assumed in the training data set, the network modeling itself will be physics-guided. The method aims at obtaining a trained generative network $G$ able to reproduce multiple realistic and equiprobable facies realizations fitting ${\mathbf{d}}_{\mathbf{o}\mathbf{b}\mathbf{s}}$.

The training of the W-Net GAN accounts for the adversarial learning between ${D}^{W}$ and $G$. At each epoch, we first sample a random seismic data realization (${\mathbf{x}}_{2}$) for each of the facies images (${\mathbf{x}}_{1}$) in the training data set. These pairs are labeled as the $real$ examples for the training of ${D}^{W}$. We further define $fake$ training samples by coupling the seismic realizations ${\mathbf{x}}_{2}$ with facies images generated at that epoch by $G$ ($G(\mathbf{z})$). Therefore, ${D}^{W}$ is trained to maximize the scores ${D}_{F}$, ${D}_{J}$ when the $real$ sample is used (${\mathbf{x}}_{1},{\mathbf{x}}_{2}$) and to minimize ${D}_{F}$ and ${D}_{J}$ when the input data is the $fake$ sample ($G\left(\mathbf{z}\right),{\mathbf{x}}_{2}$). As we are using only real samples for the seismic data domain, we exclude it from the adversarial training, imposing the maximization of ${D}_{S}$ in both cases. We formalize the discriminator loss as follows:

2 $${L}_{D}=-{\text{log}}\left[{D}_{F}\left({\mathbf{x}}_{1}\right)\right]-{\text{log}}\left[{D}_{J}\left({\mathbf{x}}_{1},{\mathbf{x}}_{2}\right)\right]-{\text{log}}\left[{D}_{S}\left({\mathbf{x}}_{2}\right)\right] -{\text{log}}\left[1-\left({D}_{F}\left({\text{G}}\left(\mathbf{z}\right)\right)\right)\right]-{\text{log}}\left[1-\left({D}_{J}\left({\text{G}}\left(\mathbf{z}\right),{\mathbf{x}}_{2}\right)\right)\right]$$

The first three terms of Eq. (2) represent the sample loss components for the $real$ samples for each score, while the last two refer to the $fake$ samples. Minimizing ${L}_{D}$ allows ${D}^{W}$ to learn the marginal and joint distributions of the facies and seismic patterns. The training of $G$ aims to maximize ${D}_{F}$ and ${D}_{J}$ using the generated pair of facies and ${\mathbf{d}}_{\mathbf{o}\mathbf{b}\mathbf{s}}$. In this case, we use the score ${D}_{J}$(${\text{G}}\left(\mathbf{z}\right),{\mathbf{d}}_{\mathbf{o}\mathbf{b}\mathbf{s}}$) to evaluate the probability of having that facies pattern, given the observed seismic data, while ${D}_{F}\left({\text{G}}\left(\mathbf{z}\right)\right)$ reinforces the geological accuracy of the generated patterns. The latter is equivalent to a discriminator’s score for conventional GANs: the score evaluates the geological accuracy but decreases when the ${\text{G}}\left(\mathbf{z}\right)$ variance is small with respect to that of the prior distribution. This condition occurs when the generated distributions start converging toward the inversion solutions. To avoid this conflict we progressively reduce the weight of the ${D}_{F}\left({\text{G}}\left(\mathbf{z}\right)\right)$ over the total loss of $G$, proportionally to half of the facies convergence to ${\mathbf{d}}_{\mathbf{o}\mathbf{b}\mathbf{s}}$. We hence formalize the loss of $G$ as

3 $${L}_{G}=-{\text{log}}\left[{D}_{F}\left({\text{G}}\left(\mathbf{z}\right)\right)+\frac{1}{2}{D}_{J}\left({\text{G}}\left({\text{z}}\right),{\mathbf{d}}_{\mathbf{o}\mathbf{b}\mathbf{s}}\right)\right]-{\text{log}}\left[{D}_{J}\left({\text{G}}\left({\text{z}}\right),{\mathbf{d}}_{\mathbf{o}\mathbf{b}\mathbf{s}}\right)\right]$$

The minimization of ${L}_{G}$ in Eq. (3) aims at the condition for which if ${D}_{J}\left({\text{G}}\left({\text{z}}\right),{\mathbf{d}}_{\mathbf{o}\mathbf{b}\mathbf{s}}\right)=1$ (i.e., perfect match), ${D}_{F}\left({\text{G}}\left(\mathbf{z}\right)\right)+\frac{1}{2}{D}_{J}\left({\text{G}}\left({\text{z}}\right),{\mathbf{d}}_{\mathbf{o}\mathbf{b}\mathbf{s}}\right)\ge 0.5$. To condition the modeling to well-log data, we include another term in ${L}_{G}$. The content loss (${L}_{C}$) is defined as the L2-norm between the observed data and the collocated predicted values. Equation (3) can hence be rewritten as

4 $${L}_{G}=-{\text{log}}\left[{D}_{F}\left({\text{G}}\left(\mathbf{z}\right)\right)+\frac{1}{2}{D}_{J}\left({\text{G}}\left({\text{z}}\right),{\mathbf{d}}_{\mathbf{o}\mathbf{b}\mathbf{s}}\right)\right]-{\text{log}}\left[{D}_{J}\left({\text{G}}\left({\text{z}}\right),{\mathbf{d}}_{\mathbf{o}\mathbf{b}\mathbf{s}}\right)\right]+\beta *{L}_{C},$$

where $\beta$ is a weight regulating the contribution of ${L}_{C}$ on ${L}_{G}$. The W-Net GAN’s parameters are trained using the ADAM optimizer^45^ with a learning rate step decay per epoch to refine the training at each epoch. In the proposed W-GAN, the number of epochs, the initial learning rate and learning rate decay rate, and the factor $\beta$, are the only hyperparameters to calibrate.

### Code implementation

The proposed seismic inversion algorithm was implemented in Python (version 3.9). We used PyTorch with CUDA (versions 1.13 and 11.7) for the W-Net GAN model and training code. The training was conducted on a computer running Windows 11 operating system, with Intel® Core™ i7-8750H CPU and NVIDIA® GeForce™ GTX 1060. The average training time of the W-Net GAN was approximately 45 s per epoch for all the synthetic application examples, and 55 s per epoch for the real application example.

## Application examples

We apply the proposed seismic inversion methodology to 2-D synthetic and real case applications. The first case is used as proof of concept and validation of the W-GAN seismic inversion, while the real case example assesses the performance of the proposed method in real noise conditions.

### Synthetic case application

**Figure 2:**
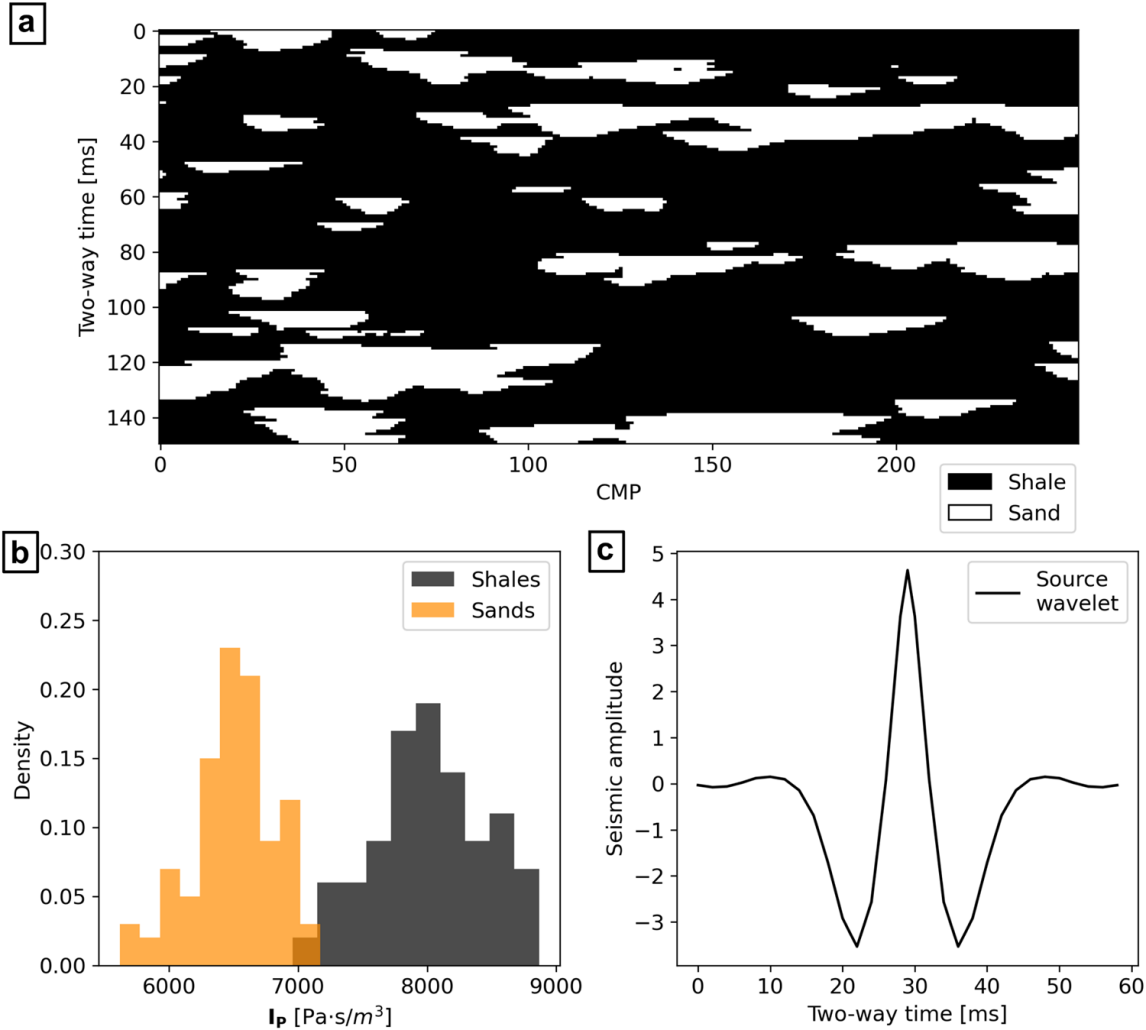
Prior training data used for the synthetic case application. (**a**) 2-D section of the conceptual geological model; (**b**) facies-dependent ${\mathbf{I}}_{\mathbf{P}}$ distributions; (**c**) wavelet used to generate the seismic data.

We assumed a synthetic depositional sequence of channel sand bodies in a shale background. Following the proposed workflow, we first represented this scenario using a conceptual 3-D geological model (Fig. 2a). Two synthetic facies-dependent ${\mathbf{I}}_{\mathbf{P}}$ distributions were defined, simulating direct observations from well-log data (Fig. 2b). The ${\mathbf{I}}_{\mathbf{P}}$ values were sampled from normal distributions with $\mu =$ 6500 Pa s/m^3^ and $\mu =$ 8000 Pa s/m^3^ for sand and shales, respectively, and both with standard deviation of 500 Pa s/m^3^. The spatial continuity pattern of ${\mathbf{I}}_{\mathbf{P}}$ was defined by a variogram model with a spherical structure, no nugget effect, and horizontal range of 80 m for both facies. The vertical range value is set to 50 ms and 80 ms for sand and shale, respectively. We generated a training data set of facies and corresponding seismic data honoring these parameters. The resulting dataset accounts for 3000 MPS simulations of facies, populated on average by 79% ± 3% of shales and 21% ± 3% of sands. Each facies realization is coupled with 16 conditioned DSS realizations of ${\mathbf{I}}_{\mathbf{P}}$, then used to compute the corresponding fullstack seismic data, using a given known wavelet (Fig. 2c). We assumed no uncertainty in the wavelet estimation.

**Figure 3:**
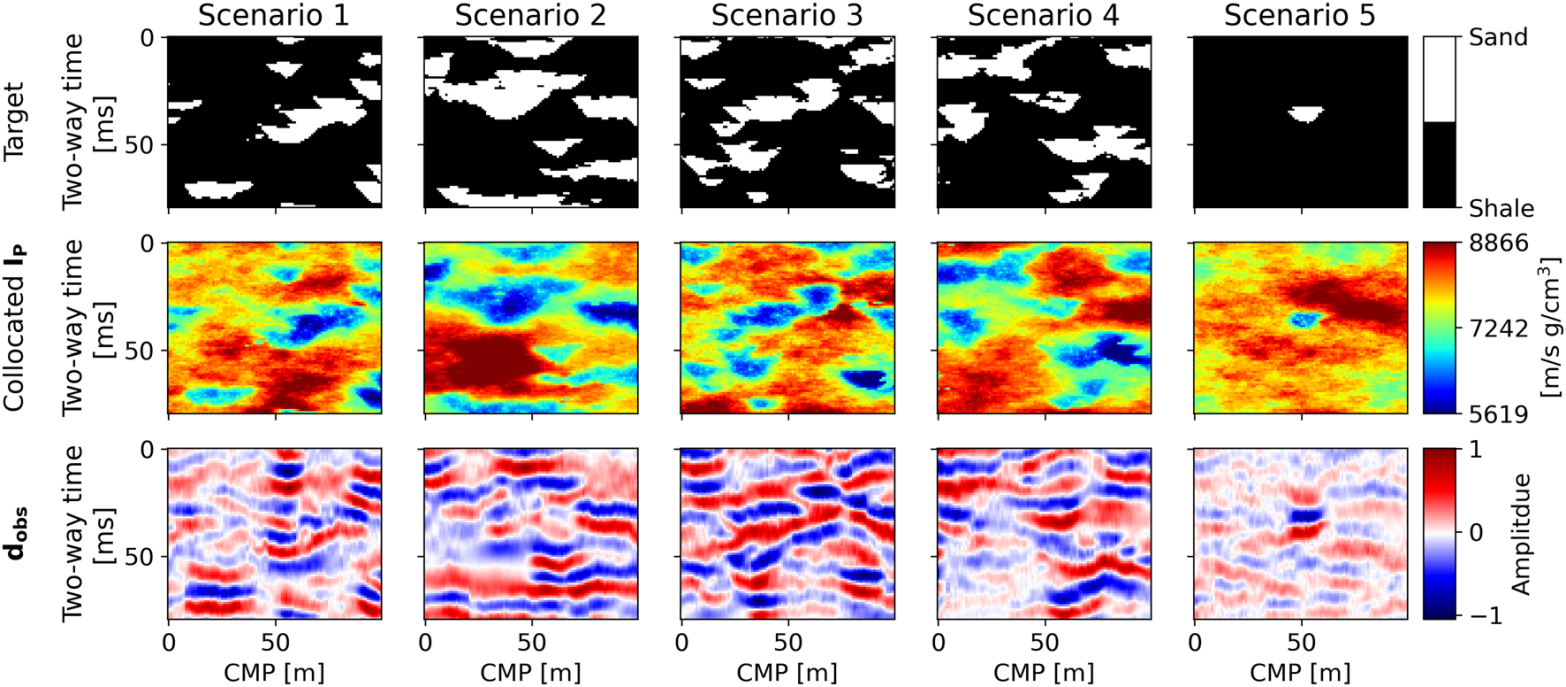
Test set used for the synthetic case application. The target facies distribution of Scenario 5 was arbitrarily generated to reproduce a sample outside the prior distribution.

Four additional facies realizations, together with the corresponding ${\mathbf{I}}_{\mathbf{P}}$ and fullstack seismic data, were also generated through the process described above and used as test scenarios. Using the fullstack seismic data as ${\mathbf{d}}_{\mathbf{o}\mathbf{b}\mathbf{s}}$, the facies patterns represent the target of our inversion (i.e., the facies we want to predict). A fifth test scenario, with an arbitrary spatial pattern, different from the other MPS simulations, was also considered. The corresponding ${\mathbf{d}}_{\mathbf{o}\mathbf{b}\mathbf{s}}$ were calculated following the description above. This scenario is designed to test the W-Net GAN in the case of training with a biased or poorly informed prior. The five test scenarios are shown in Fig. 3. For each seismic inversion, the W-Net GAN was trained for 500 epochs, using an initial learning rate of 1e-3 and a step decay rate of 50% every 50 epochs.

**Figure 4:**
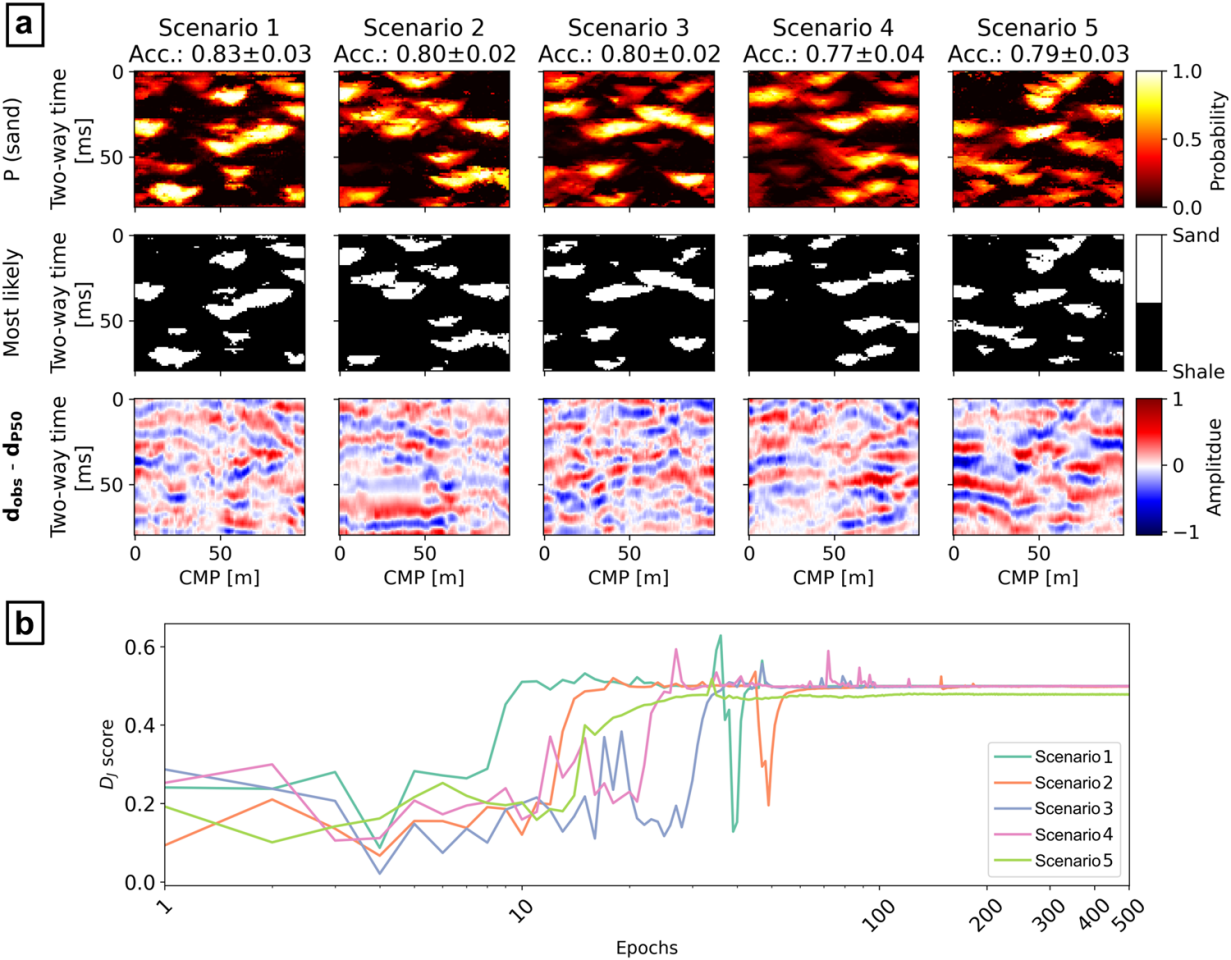
Results of the inversions for the five test scenarios of the synthetic application examples: (**a**) probability of sands, most likely facies, and data misfit between the observed data (${\mathbf{d}}_{\mathbf{o}\mathbf{b}\mathbf{s}}$) and the average seismic from the realizations’ ensemble (${\mathbf{d}}_{\mathbf{P}50}$); (**b**) ${{\varvec{D}}}_{{\varvec{J}}}$ score evolution with epochs (horizontal axis is in logarithmic scale).

The result of the inversion shows that the W-Net GAN is able to invert ${\mathbf{d}}_{\mathbf{o}\mathbf{b}\mathbf{s}}$ and produce facies models according to the given parameters and physics model. A summary of the inversion results is given in Fig. 4a. From the analysis of the evolution of the ${D}_{J}$ scores of the generated images, the adversarial training tends to stabilize before epoch 100, where the inversion results start matching ${\mathbf{d}}_{\mathbf{o}\mathbf{b}\mathbf{s}}$ (Fig. 4b). Nonetheless, the network keeps refining the features learned, and the facies realizations are consequently improved, after this point.

The facies generated by the trained $G$ were used as realizations to approximate the posterior distribution (i.e., the target) of the seismic inverse problem. From an ensemble of 500 facies realizations, a probability map of sands and the map of most likely facies per inversion grid node were calculated (Fig. 4a). Each facies realization match the target with an average accuracy spanning from 77% (for test Scenario 4) to 85% (Scenario 1). The sand probability maps resulting from the five scenarios generally capture most of the sand bodies of the target, although they show larger uncertainties in regions where the ${\mathbf{I}}_{\mathbf{P}}$ contrasts are smaller (Fig. 3). This is also reflected in the approximation by the most-likely facies models of the corresponding target. Using these distributions to condition 30 ${\mathbf{I}}_{\mathbf{P}}$ geostatistical realizations, we computed the corresponding average seismic (${\mathbf{d}}_{\mathbf{P}50}$). The seismic data misfit between ${\mathbf{d}}_{\mathbf{P}50}$ and ${\mathbf{d}}_{\mathbf{o}\mathbf{b}\mathbf{s}}$ is given in Fig. 4a. These images enhance the areas of larger misclassification. The results obtained for Scenario 5 show a clear overestimation of sand with respect to the target facies image. Each realization generated by the trained $G$ is populated, on average, by 78% ± 4% of shales and 22% ± 3% of sands, similar to the other cases’ realizations and to the training data. Nonetheless, the ensemble of the generated images approximates the target, representing the highest sand probabilities in correspondence with the target (Fig. 4a).

**Figure 5:**
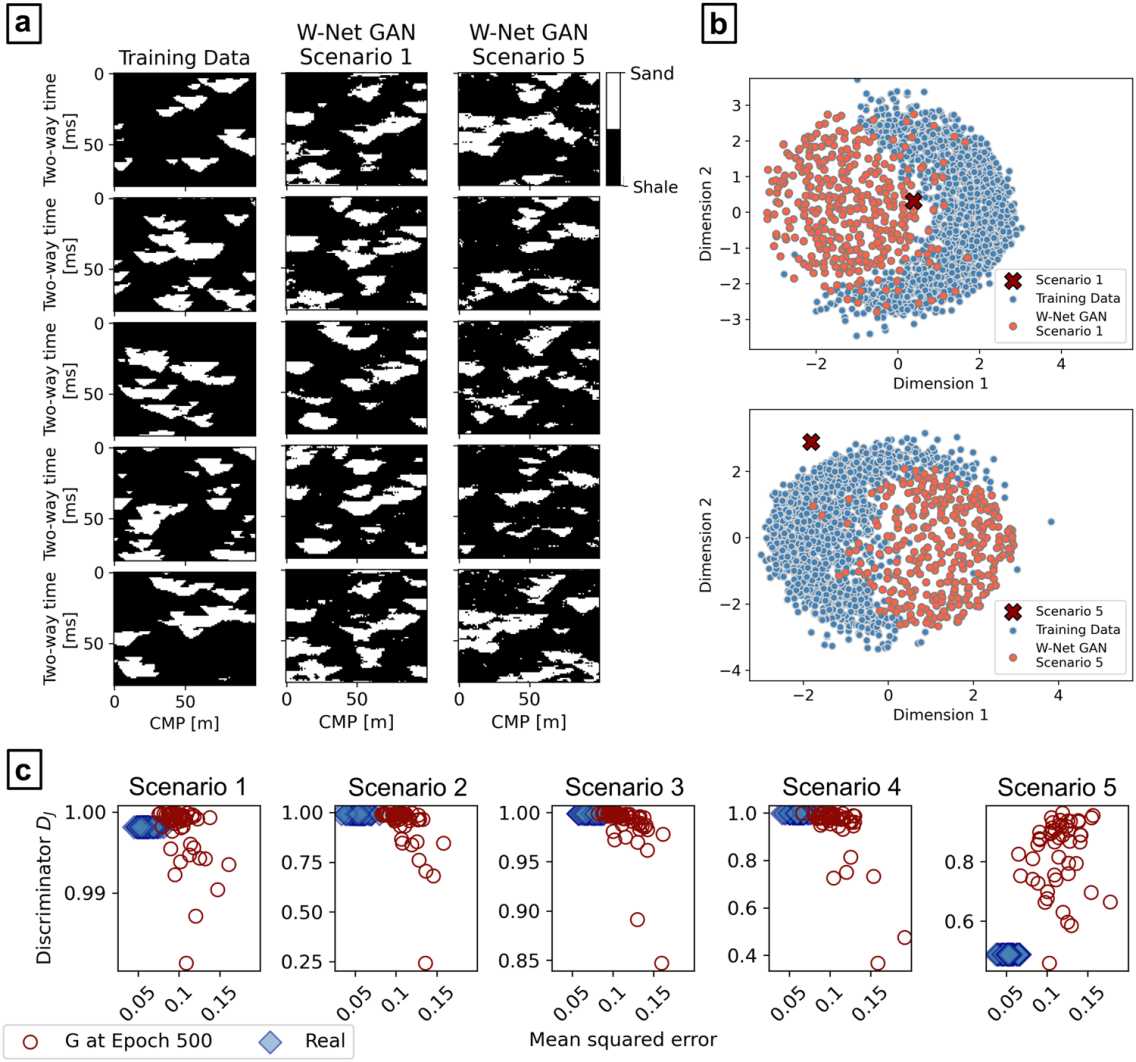
Evaluation of the W-Net GAN realizations: (**a**) comparison with the training facies samples; (**b**) 2-D MDS representation of the Hausdorff distances between training (blue circles), predicted (red circles), and target facies (red crosses); (**c**) comparison of the ${{\text{D}}}_{{\text{J}}}$ scores with the MSE between seismic realizations conditioned of the predicted facies and ${{\text{d}}}_{{\text{obs}}}$.

The single facies realizations obtained from the trained $G$ tend to reproduce well the patterns of the training data. Figure 5a shows a visual comparison of the facies realizations obtained for Scenario 1 and Scenario 5 with those of the training data set. Nonetheless, some realizations show noise or small irregularities in the facies patterns, especially in Scenario 5. We further assess the variance of these realizations with respect to the training data and their convergence to the target facies, by representing the pairwise Hausdorff distances between all the training, the target and generated samples in two dimensions, through multi-dimensional scaling (MDS)^46^. The MDS plots project the facies patterns distances, highlighting the convergence of the patterns with the inversion target (red crosses). In Fig. 5b we analyze both the results for Scenario 1 and Scenario 5, comparing 1000 generated facies realizations with the same number of training images and the target facies pattern. In both MDS plots, the data points of the generated images cluster in regions close to those of the training images, confirming that the facies patterns are similar to the prior data. These data points tend to cluster in specific regions of the MDS space or relatively smaller variance, identifying the solutions’ regions predicted by the W-Net GAN. For Scenario 1, the solutions converge correctly toward the target. The MDS plot for Scenario 5 highlights the distance of the target facies image from the training data and confirms the observations described previously on the W-Net GAN predicted facies distribution.

Finally, we evaluated whether the ${D}_{J}$ scores obtained from the trained ${D}^{W}$ (i.e., the goodness of fit of the facies to ${\mathbf{d}}_{\mathbf{o}\mathbf{b}\mathbf{s}}$**,** given the learned physics and data) provide a measure that is comparable to the mean squared error (MSE) between predicted and observed seismic data. For this purpose, we considered 50 facies realizations generated by the trained $G$, together with their corresponding ${D}_{J}$ scores. For each facies, we computed a corresponding seismic reflection data using from a single ${\mathbf{I}}_{\mathbf{P}}$ conditioned realization and computed their MSE values relative to ${\mathbf{d}}_{\mathbf{o}\mathbf{b}\mathbf{s}}$. The test was repeated for each scenario, plotting MSE values and ${D}_{J}$ scores as scatter plot (red empty circles in Fig. 5c). The cluster of points identifies an inverse correlation between ${D}_{J}$ and the MSE distance, with an average Pearson’s correlation coefficient of $-0.6\pm 0.18$. This correlation is absent for Scenario 5, where the points have larger variance. Although the coefficients of the correlation varied significantly with each training, case study and data uncertainty, the linear correlation was found to be statistically significant (p-value of 0.017). We repeated the same test using the target facies distributions (Fig. 3). For each facies distribution we simulated 50 seismic reflection data realizations and evaluated the corresponding ${D}_{J}$ scores, using the trained ${D}^{W}$ of the relative test scenario. Moreover, we calculated the MSE distances of each seismic data realization from the actual ${\mathbf{d}}_{\mathbf{o}\mathbf{b}\mathbf{s}}$ (Fig. 3). The ensemble of 50 ${D}_{J}$ scores are plotted against the corresponding MSE distances as blue diamonds in Fig. 5c. We refer to this cluster of points as *Real* data points. For each scenario, except for Scenario 5, the ${D}_{J}$ scores are close to one (~ 0.999). Their distribution does not show any variability due to the different seismic data realizations used as input to ${D}^{W}$. Contrarily, as the variance of the seismic reflection responses in the realizations is dependent on the spatial uncertainty of ${\mathbf{I}}_{\mathbf{P}}$, the MSE distances obtained show a distribution, accordingly. A comparison between the seismic inversion predictions and the *Real* data points highlights how the $G$ realizations with larger ${D}_{J}$ scores plot close to the *Real* samples, while their MSE variances are comparable to the reference. Nonetheless, the MSE values of the $G$ predictions present a shift toward relatively higher values in all the five Scenarios. The lower ${D}_{J}$ scores for the *Real* distribution are particularly lower than those predicted; this is an expected behavior as the real facies image does not belong to the training data distribution.

### Real case application

**Figure 6:**
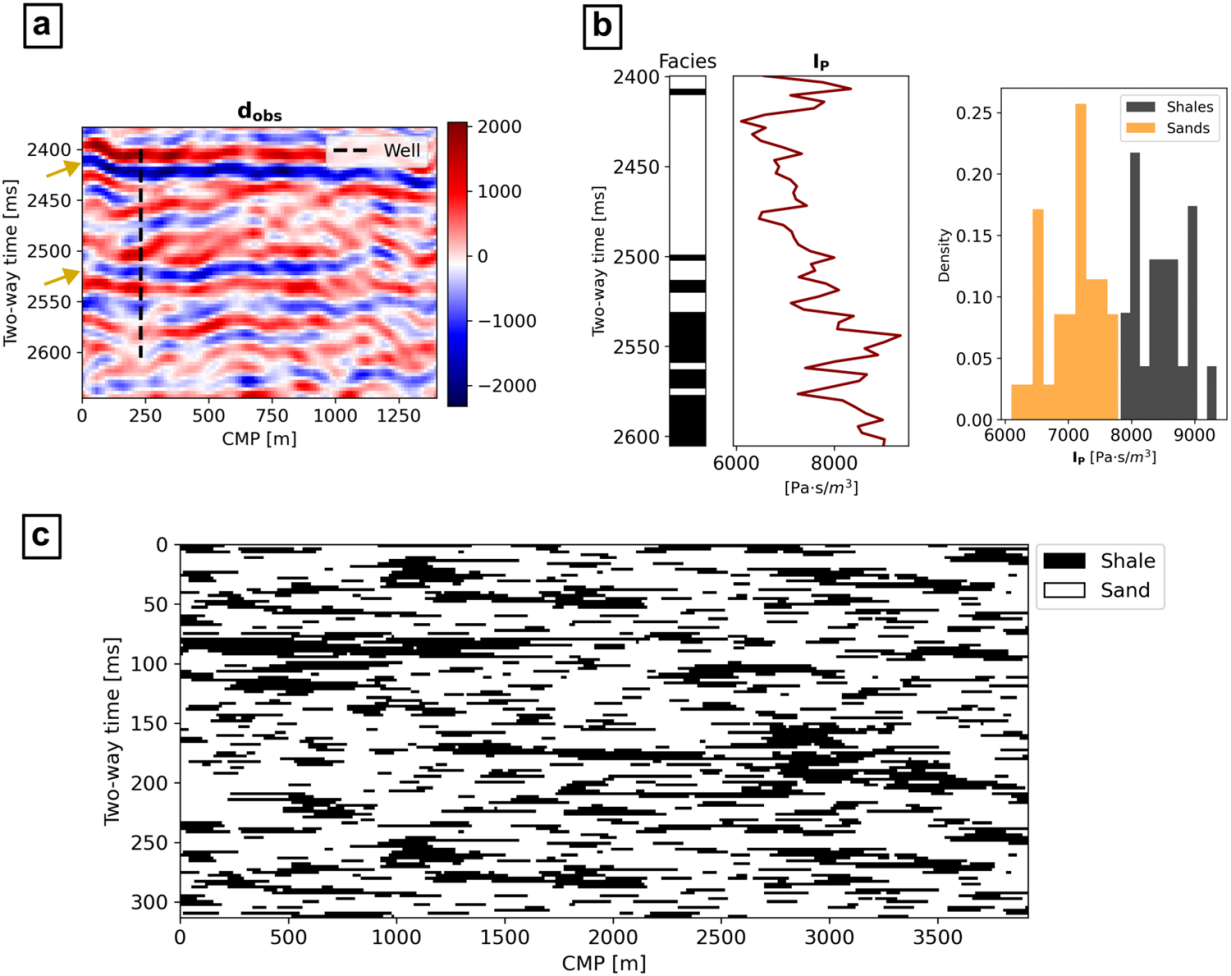
Observed data and prior used for the inversion; (**a**) observed seismic reflection data from the Norne Field and location of the well data. The yellow arrows indicate the location of two major seismic reflection events; (**b**) well log data; (**c**) 2-D vertical section of the conceptual geological model used for the training data set’s facies realizations.

We selected a 2-D fullstack seismic section extracted from a 3-D seismic survey acquired in the Norne field, surveying a shallow marine to fluvio-deltaic sedimentary sequence (Fig. 6a). The survey extends for 1400 m laterally, covering a vertical interval of 267 ms two-way time (TWT); the inversion grid defined has 109 × 75 cells. A well crosses the survey path, from which we extracted the facies profile and the corresponding ${\mathbf{I}}_{\mathbf{P}}$ distributions (Fig. 6b). The facies identified in the area were defined as sands and shales. Given the available geological knowledge^47,48^, we set up a prior 3-D geological model describing the spatial features of the expected intercalation of shale layers and sand bodies. The latter were described as a combination of more tabular bodies mixed with channel-like shapes. The ${\mathbf{I}}_{\mathbf{P}}$ spatial uncertainty is described by a variogram model fitting the experimental well-log data (i.e., spherical variogram model; no nugget effect; range of 13 ms and 26 m for vertical and horizontal directions, respectively). The source wavelet was initially extracted from the original survey and finely tuned so that the synthetic seismic data calculated from the ${\mathbf{I}}_{\mathbf{P}}$-logs would fit ${\mathbf{d}}_{\mathbf{o}\mathbf{b}\mathbf{s}}$. We then generated a data set of 3000 facies patterns and 16 seismic reflection responses each. The facies images in the training data are populated by 26% by shales and 74% by sands ($\sigma$ = 4%). We ran two seismic inversions using the well data for blind-well testing (unconditioned case) and conditioning the realizations on the well profiles (conditioned case), using Eqs. (3) and (4) for the training of $G$. We set the same hyperparameters used for the synthetic case in both inversions: 500 training epochs and an initial learning rate of 1e-3 with a step decay of 50% per 50 epochs. For the conditioned case, we set $\beta$ = 1 [Eq. (4)].

**Figure 7:**
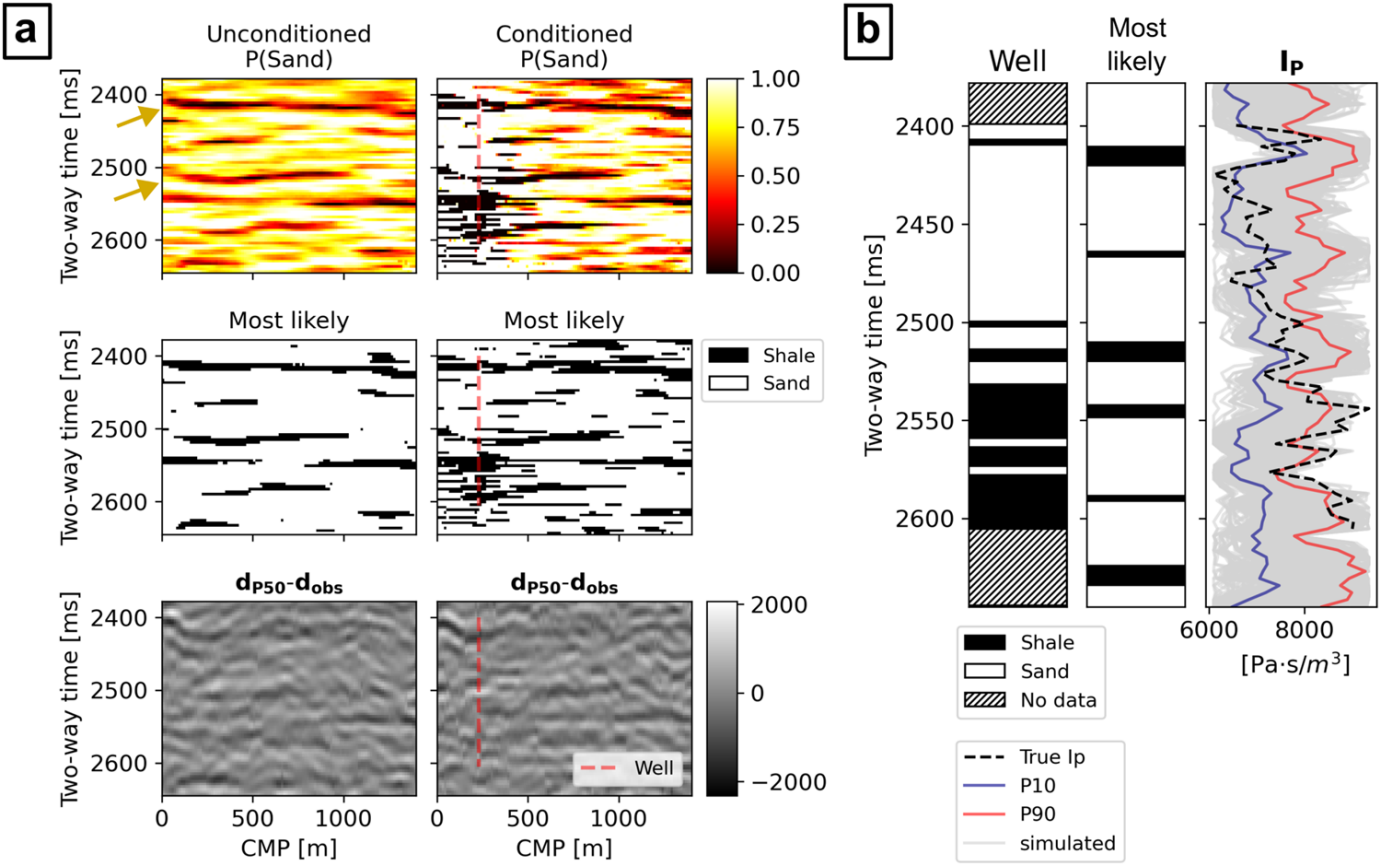
Results of the seismic inversions; (**a**) sand probability, most likely facies, and average seismic data misfit, for both conditioned and unconditioned cases. The yellow arrows indicate the location of the two major reflections event in ${{\text{d}}}_{{\text{obs}}}$ (Fig. 6a); (**b**) comparison of the well-log data with the collocated predicted facies and corresponding conditioned ${{\text{I}}}_{{\text{P}}}$ distribution.

**Figure 8:**
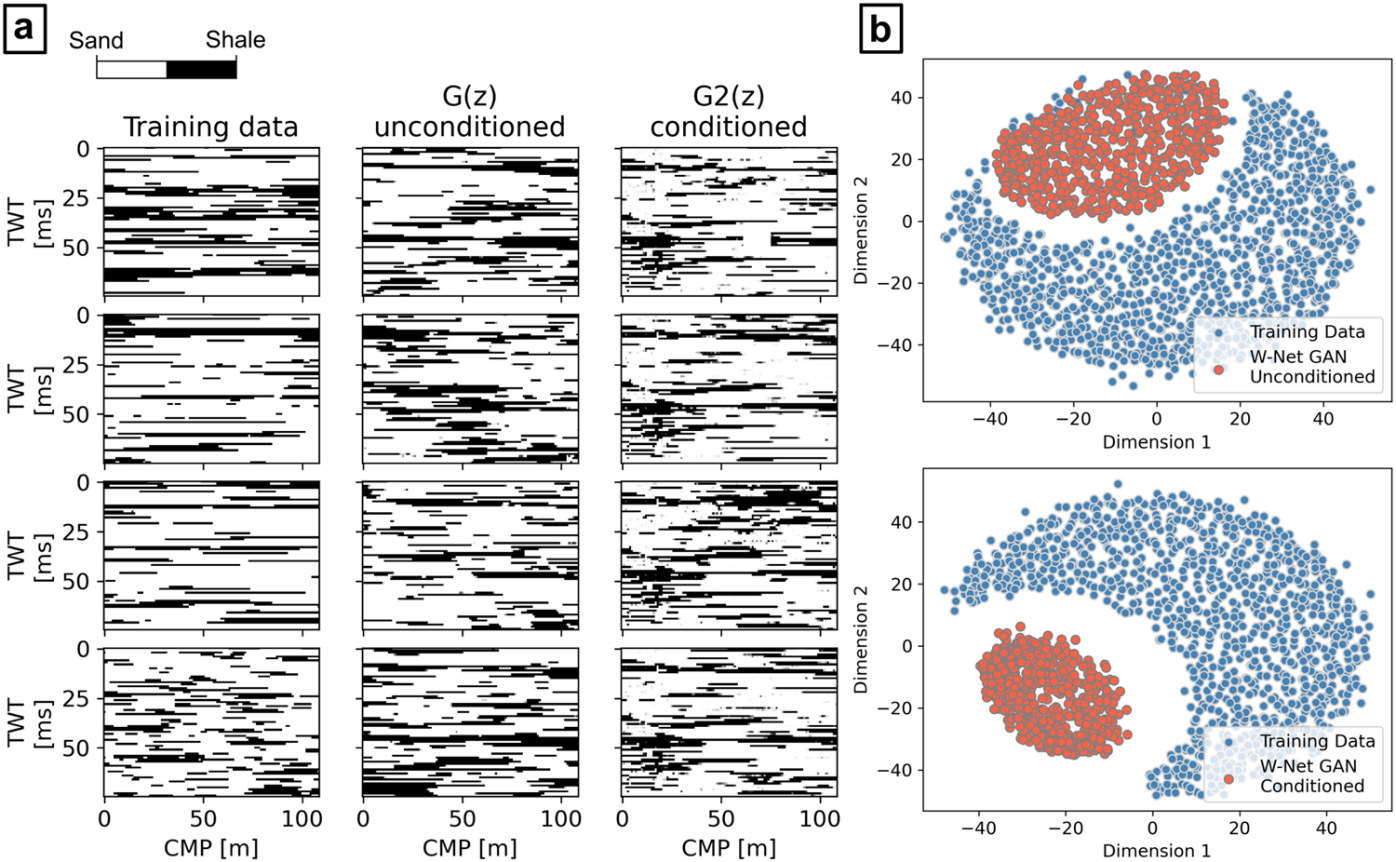
Evaluation of the W-Net GAN realizations after training, for both unconditioned and conditioned cases. (**a**) Visual comparison with the training data; (**b**) 2-D MDS representation of the Hausdorff distances between training, predicted, and target facies.

An ensemble of 500 facies generated by $G$ after the two inversions was used to compute the probability of sands over shales, the corresponding most likely facies, and the difference between the average expected seismic from the predicted facies and ${\mathbf{d}}_{\mathbf{o}\mathbf{b}\mathbf{s}}$ (Fig. 7a). Except for the well-log location, the solutions retrieved by the conditioned and the unconditioned cases match in terms of shale layers identified and their morphology. For example, laterally continuous shale layers match two main horizontal seismic reflection events in ${\mathbf{d}}_{\mathbf{o}\mathbf{b}\mathbf{s}}$, at approximately 2425 ms and 2525 ms TWT, for both cases. This is visible in both probability distribution models and most likely model in Fig. 7a. The locations of these reflections are highlighted by yellow arrows in both Figs. 6a and 7a. A third major layer of shales was also identified at 2550 ms, but in this case the most likely facies model shows a lateral truncation between 550 and 750 m (i.e., a relatively higher probability of sands was predicted). Analogously, the difference between the average expected seismic and ${\mathbf{d}}_{\mathbf{o}\mathbf{b}\mathbf{s}}$ is similar between the two cases: the average absolute difference of amplitudes is 327 ± 252 for the unconditioned case and 343 ± 253 for the conditioned one. At the well location, the conditioned facies probabilities perfectly match with the conditioning well-log data, while the unconditioned case retrieves a set of solutions with relatively large uncertainty. The comparison of the real facies profile with the collocated most likely facies predicted in the unconditioned case (Fig. 7b) indeed shows an underestimation of shales. Nonetheless, the well-log ${\mathbf{I}}_{\mathbf{P}}$ data (“True Ip” in Fig. 7b) is entirely within the range of collocated ${\mathbf{I}}_{\mathbf{P}}$ values conditioned on the predicted facies, except from one point of the simulation grid, at 2550 ms, where the predictions reproduce slightly lower ${\mathbf{I}}_{\mathbf{P}}$ values. This suggests that the facies uncertainty modeled by $G$ can be explained by the actual ${\mathbf{I}}_{\mathbf{P}}$ data uncertainty from the well-log. The morphology of the facies in the single realizations visually match quite well those of the training images (e.g., Fig. 8a) and they perfectly match the average and variance of the shale-to-sand ratio. Despite these similarities, the conditioned case results show a relatively smaller variance of the predicted model when compared to the unconditioned case in the locations away from that of the well. This effect can be interpreted by inspecting the differences between the predicted sand probability maps (Fig. 7a), as well as a two-dimensional MDS representation of the predicted facies (Fig. 8b).

## Discussion

The results obtained from both the synthetic and real application examples demonstrate that the W-Net GAN is able to invert fullstack seismic reflection data, by learning the physics-based correlation between facies and seismic data domains. Overall, the predicted facies distributions reproduce the patterns and the facies statistics of the training data set in both synthetic and real applications. Besides, the proposed method is able to account for existing direct measurements (i.e., well-log data) or predict facies in scenarios where these data are not available.

The solutions for the synthetic case applications show high accuracy of the facies realizations when compared to the inversion target. When the prior is biased or poorly informed, as demonstrated by the results in Scenario 5, the network adapts the learned features to the observed data, approximating the target scenario, although with significant errors (Figs. 4 and 5). The presence of noise in the generated images, visible in the result of Scenario 5 (Fig. 5a) can be an indication of model overfitting, or due to the inability of the network to reproduce specific target features given the training data set parameters. A possible solution to avoid noisy images can be to increase the weight of ${D}_{F}$ and reducing that of ${D}_{J}$ in the loss function of $G$ [Eqs. (3) and (4)]. Nonetheless this is likely to increase the variance of the solutions, above that induced by the ${\mathbf{I}}_{\mathbf{P}}$ uncertainty, reducing the global convergence of the method. The comparison of the ${D}_{J}$ scores with the actual seismic data misfit (MSE) in Fig. 5c shows that the predicted facies patterns reproduce a variability that is close to the range of ${\mathbf{I}}_{\mathbf{P}}$ data uncertainty. Moreover, the inverse correlation between the ${D}_{J}$ scores and the MSE and the consistency of the results obtained, are indicators that the trained ${D}^{W}$ is indeed able to provide a physics-dependent evaluation of the goodness of fit of the generated facies to ${\mathbf{d}}_{\mathbf{o}\mathbf{b}\mathbf{s}}$. The relatively larger MSE values showed by the predicted facies (Fig. 5c) may be explained by the presence of additional modeling uncertainty, which can be reduced by improving specific architecture’s parameters (e.g., number of layers, kernel sizes, or padding) or the training hyperparameters. On the other hand, the blind-well test for the unconditioned real case application (Fig. 7b) indicates that such uncertainty is negligible for this case.

The results obtained for the real case applications confirm analogous performances of the network. The probability maps of sands retrieved from the conditioned and the unconditioned case are comparable and reproduce major facies changes in correspondence to the main seismic reflection events. The use of local constraints (i.e., localized well-log facies profiles) conditions the generation of facies patterns also away from the well location. This did not affect the solutions in terms of seismic data misfit but significantly reduced the global uncertainty ranges. It is not possible to evaluate whether these solutions are more or less accurate than the unconditioned case as the true Ip and facies fields are unknown. Other methods such as the use of a U-Net generative network^37^ or the integration of context expansion^29^ can be adopted to priorly control the influence of the wells on the generated images.

In the proposed method, the spatial resolution of facies and corresponding ${\mathbf{I}}_{\mathbf{P}}$ distributions represented in the training data set depends on the geological information and the available well-log data. Nonetheless, the lateral accuracy of the predicted facies is limited to the seismic data spatial resolution, while the vertical resolution depends on the grid cell size in this direction. In the examples shown here we consider litho-fluid facies as geological formations with similar elastic responses at the seismic scale. For example, the low resolution of ${\mathbf{d}}_{\mathbf{o}\mathbf{b}\mathbf{s}}$ for the real case does not allow to predict the distribution of thin shale layers represented in the training data with large accuracy but approximate a probability distribution. This represents a well-known limitation of seismic data inversion^7^. Another important aspect of the proposed applications is that we assume no uncertainty in the physics forward model used. This should be considered in more complex real scenarios, e.g., by simulating seismic data realizations reflecting this uncertainty in the training data set. Another possibility is to further explore the parameters space of the generative network by means of an inference method^26,40,42,49^ and refine the range of solutions predicted. These suggested applications would require further related studies to assess the specific uncertainty representation abilities of the W-Net GAN.

We have demonstrated that our neural network is capable of learning data distributions and modeling complex, nonlinear relationships in seismic data inversion. Beyond our current applications, we believe this framework can be adapted for other geophysical data inversions and the prediction of various Earth properties. This may require relatively small adjustment to the network architecture and the representation of relevant prior knowledge in the training data set, which may be topics for future research projects.

## Conclusions

We propose the W-Net GAN as a physics-based GAN for the direct inference of accurate facies distributions from fullstack seismic data. The proposed training algorithm aims at obtaining a discriminator (${D}^{W}$) that is able to recognize the features of seismic data and represents its correlation to the facies domain. This is done using a multivariate prior representing facies and seismic data features, as well as their corresponding physics-based correlation. The ${D}^{W}$ network is designed to have a three-branched architecture, each encoding the information from the two domains into three different scores, representing the marginal probabilities of facies and seismic and their joint bivariate probability. Using the observed seismic data ${\mathbf{d}}_{\mathbf{o}\mathbf{b}\mathbf{s}}$ as input to ${D}^{W}$, we can hence evaluate the facies generated by an unconditional generative network in terms of geological accuracy and fit to the seismic data. The trained generator reproduces equiprobable realizations of accurate facies patterns fitting ${\mathbf{d}}_{\mathbf{o}\mathbf{b}\mathbf{s}}$, hence approximating a non-parametrical posterior distribution of the solutions. Compared to conventional methods using GANs to parametrize a prior distribution of facies^26,40,42,48^, the W-Net GAN presents two main advantages: the ability to invert seismic data in a single training step while accounting for the spatial uncertainty of facies-dependent continuous properties in the retrieved distribution. The method was tested successfully on a synthetic scenario using 5 test scenarios, one of which simulated a case of inversion with biased prior. The retrieved solutions show an average accuracy of 80% and demonstrate that approximate solutions are possible for cases not represented in the prior, although presenting biases due to the learned subsurface parameters. We also demonstrated the application of the W-Net GAN by inverting a real 2-D fullstack seismic section, using the available well-log profiles both as local conditioning data and for blind-well testing. The high quality of the retrieved solutions in terms of accuracy and uncertainty reproduction validate the applicability of the method for different complex scenarios.

### Supplementary Information

Supplementary Figures.

## Data Availability

The python codes for the W-Net GAN inversion algorithm and data for the applications presented in this paper are available at https://github.com/romiele/W-NetGAN.
