# Supplementary material for: Physics-informed W-Net GAN for the direct stochastic inversion of fullstack seismic data into facies models
Source: Sci Rep. 2024 Mar 1;14:5122. doi: 10.1038/s41598-024-55683-5 (PMC10907746; doi:10.1038/s41598-024-55683-5)
Supplement: Supplementary file 1 — Supplementary Figures. [file 41598_2024_55683_MOESM1_ESM.pdf]

Supplementary Information

Physics-informed W-Net GAN for the direct stochastic inversion of fullstack seismic data into facies models

Roberto Miele[1],*, Leonardo Azevedo[1]

[1]CERENA, Department of Energy and Mineral Resources Engineering, Avenida Rovisco Pais 1, Lisboa, 1049-001, Portugal

*roberto.miele@tecnico.ulisboa.pt

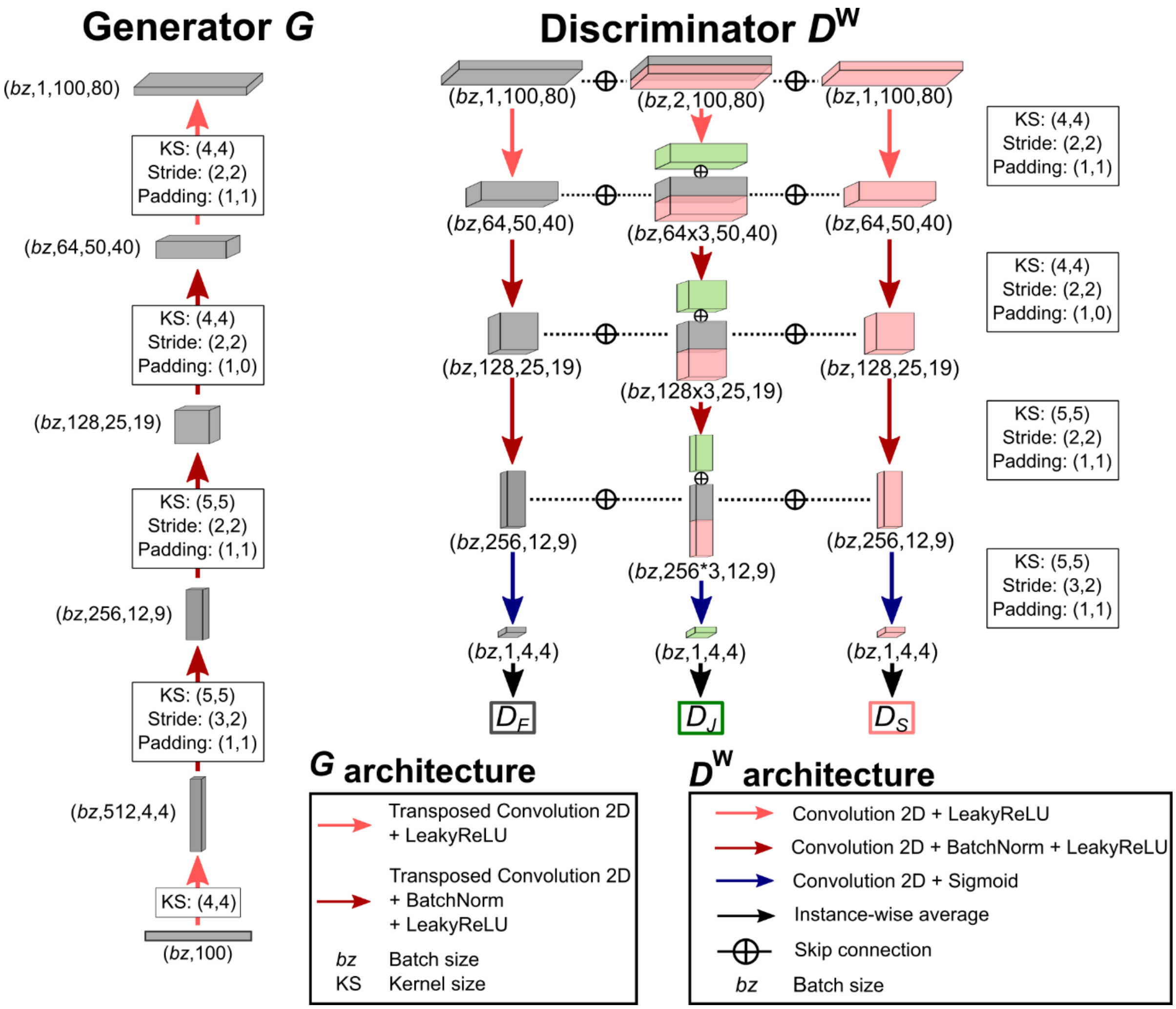

Figure S1: architecture of the W-Net GAN used for the synthetic case applications (inversion grid size 80 X 100).

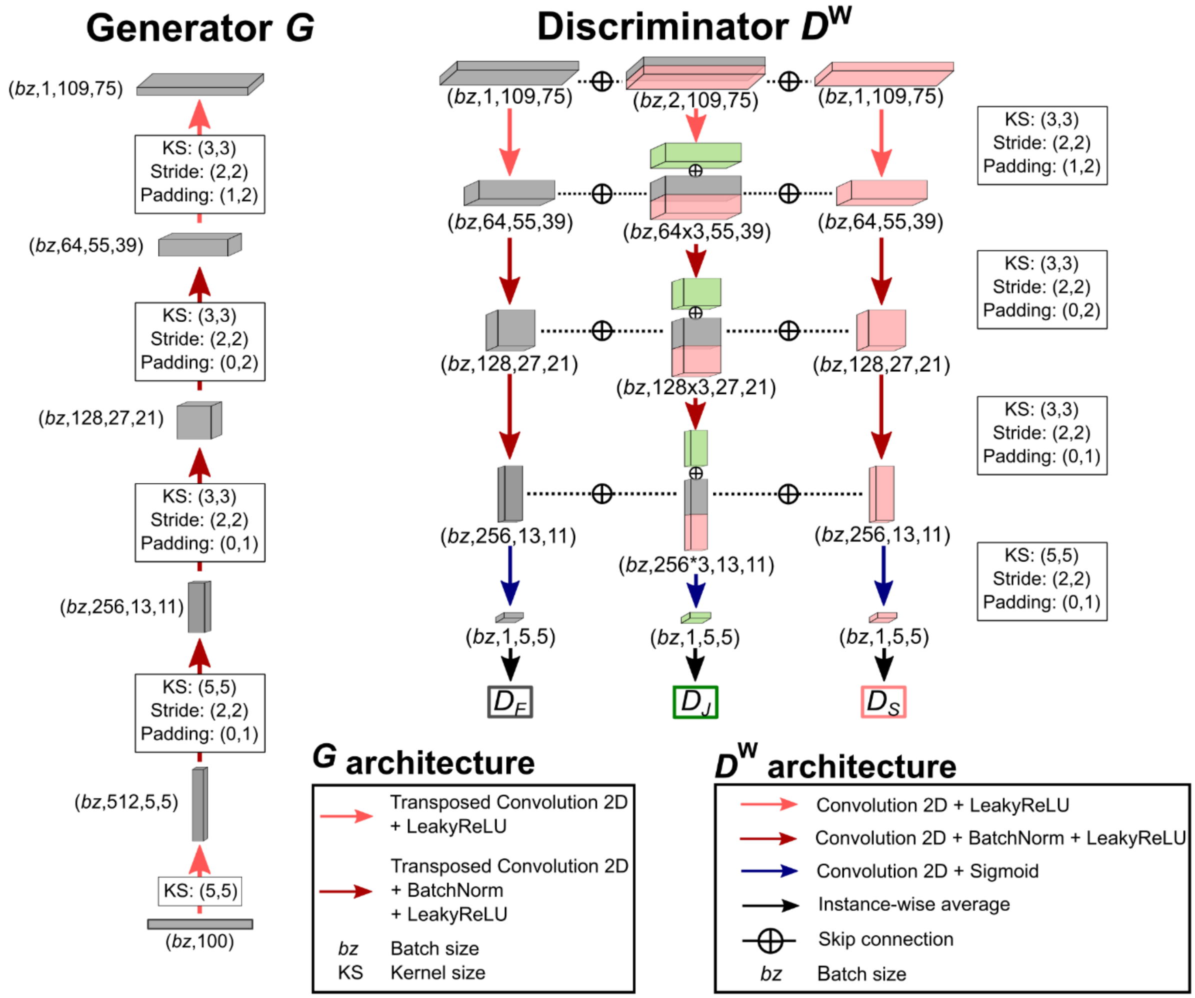

Figure S 2: architecture of the W-Net GAN used for the real case application (inversion grid size 75 X 109).
